# Dynamics of leaf and spikelet primordia initiation in wheat as affected by *Ppd-1a* alleles under field conditions

**DOI:** 10.1093/jxb/ery104

**Published:** 2018-03-17

**Authors:** Helga Ochagavía, Paula Prieto, Roxana Savin, Simon Griffiths, GustavoA Slafer

**Affiliations:** 1 Department of Crop and Forest Sciences and AGROTECNIO (Center for Research in Agrotechnology), University of Lleida, Av. Rovira Roure, Lleida, Spain; 2 John Innes Centre, Norwich Research Park, Colney Lane, Norwich, UK; 3 ICREA, Catalonian Institution for Research and Advanced Studies, Spain

**Keywords:** Double ridge, flowering time, plastochron, *Ppd* genes, *Triticum aestivum*

## Abstract

Wheat adaptation is affected by *Ppd* genes, but the role of these alleles in the rates of leaf and spikelet initiation has not been properly analysed. Twelve near isogenic lines (NILs) combining *Ppd-1a* alleles from different donors introgressed in A, B, and/or D genomes were tested under field conditions during two growing seasons together with the wild type, Paragon. Leaf initiation rate was unaffected by *Ppd-1a* alleles so the final leaf number (FLN) was reduced in parallel with reductions in the duration of the vegetative phase. Spikelet primordia initiation was accelerated and consequently the effect on spikelets per spike was less than proportional to the effect on the duration of spikelet initiation. The magnitude of these effects on spikelet plastochron depended on the doses of *Ppd-1* homoeoalleles and the specific insensitivity alleles carried. Double ridge was consistently later than floral initiation, but the difference between them was not affected by *Ppd-1a* alleles. These findings have potential for selecting the best combinations from the *Ppd-1* homoeoallelic series for manipulating adaptation taking into consideration particular effects on spikelet number.

## Teaser

Wheat *Ppd-1a* alleles did not affect leaf initiation but accelerated spikelet initiation, which partially compensated for the reduction in spikelet number due to a shorter period of spikelet initiation.

## Introduction

Flowering time is critical for adaptation of wheat to the many regions in which it is grown (Braun *et al.*, 2010). Flowering time involves three major phenological phases: vegetative, early reproductive, and late reproductive phases (Slafer and Rawson, 1994*a*). During these phases, the organs that will become the main sources and sinks of the crop, whose balance will determine yield, are initiated as primordia. All leaf primordia, in addition to those that are already differentiated in the embryo of the grain (~4), are developed during the vegetative phase. All spikelet primordia are initiated during the early reproductive phase and all florets are produced within the spikelets during the late reproductive phase (Slafer *et al.*, 2015). The internal (apical) and microscopic processes determining leaf and spikelet primordia initiation seem to be co-ordinated with leaf appearance (Kirby, 1990), which in turn determines the macroscopic, external morphological stage of Haun (1973). A major difference between these organs is that whilst all leaves and spikelets initiated will develop further and will be visible in the adult plant, within each spikelet there is an indeterminate initiation of floret primordia, most of which die (i.e. their developmental progress towards becoming fertile florets at anthesis is arrested). So the dynamics of initiation of leaves and spikelets and that of florets are dramatically different. Here we will focus on the dynamics of leaf and spikelet initiation and, in a companion paper (Prieto *et al.*, 2018), we deal with the dynamics of floret initiation, floret mortality, and the determination of fertile floret number.

Dynamics of leaf and spikelet initiation are critical in determining the final numbers of leaves and spikelets, respectively, which in turn are major determinants of yield. Final leaf number (FLN) largely determines flowering time (Kirby, 1990; Hay and Kirby, 1991; Jamieson *et al.*, 1998) and therefore adaptation (Worland and Sayers, 1996; Snape *et al.*, 2001), which is critical for optimizing yield under particular growing conditions (Rajaram *et al.*, 1990; Reynolds *et al.*, 2012). After floral initiation, all spikelets, primary branches of the inflorescence, are initiated, controlling inflorescence architecture and the first component of yield through potentially affecting grains per spike (Friedman and Harder, 2004; Kellogg *et al.*, 2013; Boden *et al.*, 2015).

Immediately after sowing (with grain imbibition), the initiation of leaf primordia resumes. By the time of seedling emergence, two additional leaves have usually been initiated. This that is why the absolute minimum FLN of a wheat plant is ~6 (Hay and Kirby, 1991). After seedling emergence, the apex continues initiating more leaf primordia until floral initiation, when the FLN in the main shoot is determined (Slafer *et al.*, 2015). The rate of leaf initiation is relatively constant (e.g. Kirby *et al.*,1987; Delécolle *et al.*, 1989; Miralles *et al.*, 2001; González *et al.*, 2002), and consequently the interval of thermal time between the initiation of two consecutive primordia (the reciprocal of the rate, termed leaf plastochron; Hay and Kirby, 1991) is constant in a particular genotype and environment for all leaves. Although it is not a fixed value, frequently leaf plastochron has been reported to be ~50 ºCd per primordium (Slafer and Rawson, 1994*a*).

Starting at floral initiation, all spikelets are initiated in the apex until the initiation of the last, terminal spikelet (when the number of spikelets per spike is fixed). The first spikelets might eventually be initiated at the same rate of leaf initiation, but even in these cases most of the spikelets are initiated at a much faster rate (e.g. Kirby, 1974; Delécolle *et al.*, 1989; Miralles and Richards, 2000), at least when unsatisfied vernalization requirements do not slow down development (González *et al.*, 2002). As double ridge is the first (albeit microscopic) morphological sign that the apex is unequivocally reproductive (i.e. developing spikelets), this stage has been frequently considered as equivalent to floral initiation. However, the change from vegetative to reproductive stage, when the first spikelet primordium is initiated, normally occurs considerably earlier (Delécolle *et al.*, 1989; Evans and Blundell, 1994). Therefore, an accurate timing of floral initiation can only be determined *a posteriori*, considering the dynamics of primordia initiation and FLN (as explained below in the Materials and Methods).

A comprehensive understanding of how environmental and genetic factors modify these rates of primordia initiation is relevant for the design of better management and breeding strategies. Previous studies showed that leaf and spikelet initiation were influenced differently by environmental cues, such as temperature (if the rates are expressed ‘per day’) and daylength (Delécolle *et al.*, 1989; Evans and Blundell, 1994; Miralles and Richards, 2000), and consequently respond to changes in sowing dates (Miralles *et al.*, 2001). On the other hand, very little is known of the effects of photoperiod sensitivity genes (*Ppd-1*) on the rates of initiation of leaf and spikelet primordia. These *Ppd-1* genes play a major role in the adaptability of wheat in a broad range of environments (Worland *et al.*, 1998; Snape *et al.*, 2001; Turner *et al.*, 2005; Griffiths *et al.*, 2009; Langer *et al.*, 2014). These developmental responses to photoperiod are mainly controlled by homoeoallelic series on the short arms of chromosome 2, *Ppd-D1*, *Ppd-B1*, and *Ppd-A1* genes (Beales *et al.*, 2007), and their effects on phenology have been quantified many times (e.g. Cockram *et al.*, 2007; Fischer, 2011). Wheat can be further classified as photoperiod sensitive and insensitive (day neutral). Photoperiod-sensitive wheat varieties need exposure to long days to flower early, while insensitive wheat are day neutral and flower early under both long- and short-day conditions (Beales *et al.*, 2007). Early flowering of the insensitivity group is due to early activation of the flowering pathway (due to misexpression of *Ppd-1*) regardless of daylength (Beales *et al.*, 2007). Photoperiod-insensitive alleles are referred to with the *a* suffix and photoperiod-sensitive alleles carry the *b* suffix (McIntosh *et al.*, 2003). However, the likely effects of these genes on the rates of primordia initiation (during the phases whose duration are affected by them) have been largely ignored, mainly because of the expertise required for, and the amount of work involved in, the proper determination of these rates (see below). In a few cases, these effects were either indirectly assessed through the final number of leaves or spikelets, or studied with materials that were not isogenic lines (Scarth *et al.*, 1985; Worland *et al.*, 1998; Snape *et al.*, 2001; Whitechurch and Slafer, 2002; Matsuyama *et al.*, 2015; Digel *et al*., 2015). Finally, the very few studies that used near isogenic lines (NILs) for *Ppd* genes considered a limited source of the *Ppd* alleles. Also, in the vast majority of cases, conclusions were drawn from studies on isolated potted plants grown in controlled conditions (with severe risks when extrapolating to field canopies; Passioura, 2010; Sadras and Richards, 2014).

To the best of our knowledge, the combination of the effects of different *Ppd* genes, of sources (donors) of a particular *Ppd* allele, and of the dosage of *Ppd* genes on leaf and spikelet primordia initiation have not been analysed before. In this study, we aimed to explore under field conditions the impact of photoperiod insensitivity alleles with NILs considering different alleles, for some of them different sources, and dosage on the dynamics of primordia initiation resulting in differences in plastochron of leaves and spikelets. Furthermore, we assessed to what degree the effects of these genes on primordia initiation rates were direct or linked with their effects on other developmental processes: whether the *Ppd* effects (i) on rates of development explained part of the effects on the final number of spikelets per spike and (ii) on rates of primordia initiation were mediated through effects on the dynamics of leaf appearance (i.e. whether the effects on plastochron and phyllochron were co-ordinated).

## Materials and methods

### Field conditions, treatments, and design

Field experiments were carried out during the 2012/13 and 2013/14 growing seasons, under stress-free conditions (i.e. weeds, insects, and diseases were controlled or prevented and plots were irrigated/fertilized as required) close to Bell-lloc d’Urgell (41.63°N, 0.78°E), Catalonia, North-East Spain. The soil was classified as a complex of Calcisol petric and Calcisol haplic, following the soil classification of the FAO (1990). Both experiments (2012/13 and 2013/14 growing seasons) were sown on optimum dates for the Mediterranean region of the Ebro Valley, on 24 November 2012 and 12 November 2013, respectively. We aimed to have a density of 240 plants m^–2^ uniformly distributed. For that purpose, we sowed the plots at a rate of 300 grains m^–2^. A week after emergence, we labelled sampling areas in each plot and, when necessary, we thinned these areas by hand to have that density with very high uniformity. We counted the number of adult plants at anthesis corroborating that the plant density wanted was effectively achieved in the samples: the measured density in the sample taken at anthesis (see below) was 238 ± 5 and 247 ± 4 plants m^–2^ in the first and second growing season, respectively.

Meteorological data were recorded daily by a meteorological station from the agro-meteorological network of Catalonia located close to the experimental site. Temperatures were warmer in the second than in the first growing season, particularly the maximum temperatures during late winter and spring, February to June, when most crop growth takes place (Table 1).

**Table 1.** Monthly mean minimum and maximum temperatures and photoperiod for the first (2012/13) and second (2013/14) growing seasons, and the averaged temperatures for the six previous years of the experiment (from 2007 to 2012)

		Nov	Dec	Jan	Feb	Mar	Apr	May	Jun	Jul
Minimum temperature (ºC)	2012/13	3.5	–0.5	–0.8	–0.4	4.1	5.5	7.6	11.7	15.1
	2013/14	1.0	–0.8	2.6	1.7	3.3	8.6	9.0	14.0	15.5
	2007–2012	2.9	0.1	0.0	0.2	2.7	6.5	10.4	13.7	16.0
Maximum temperature (ºC)	2012/13	11.8	10.7	9.9	11.8	15.8	18.8	20.4	26.9	33.8
	2013/14	10.8	7.4	11.7	12.9	17.7	22.5	24.4	29.8	30.4
	2007–2012	14.1	9.6	8.8	12.9	16.7	19.8	24.8	28.7	31.2
Photoperiod (h)	2012/13	9.3	9.1	9.4	10.4	11.7	13.1	14.3	14.9	14.6
	2013/14	9.5	9.1	9.4	10.4	11.7	13.1	14.3	14.9	14.6

**Fig. 1.**
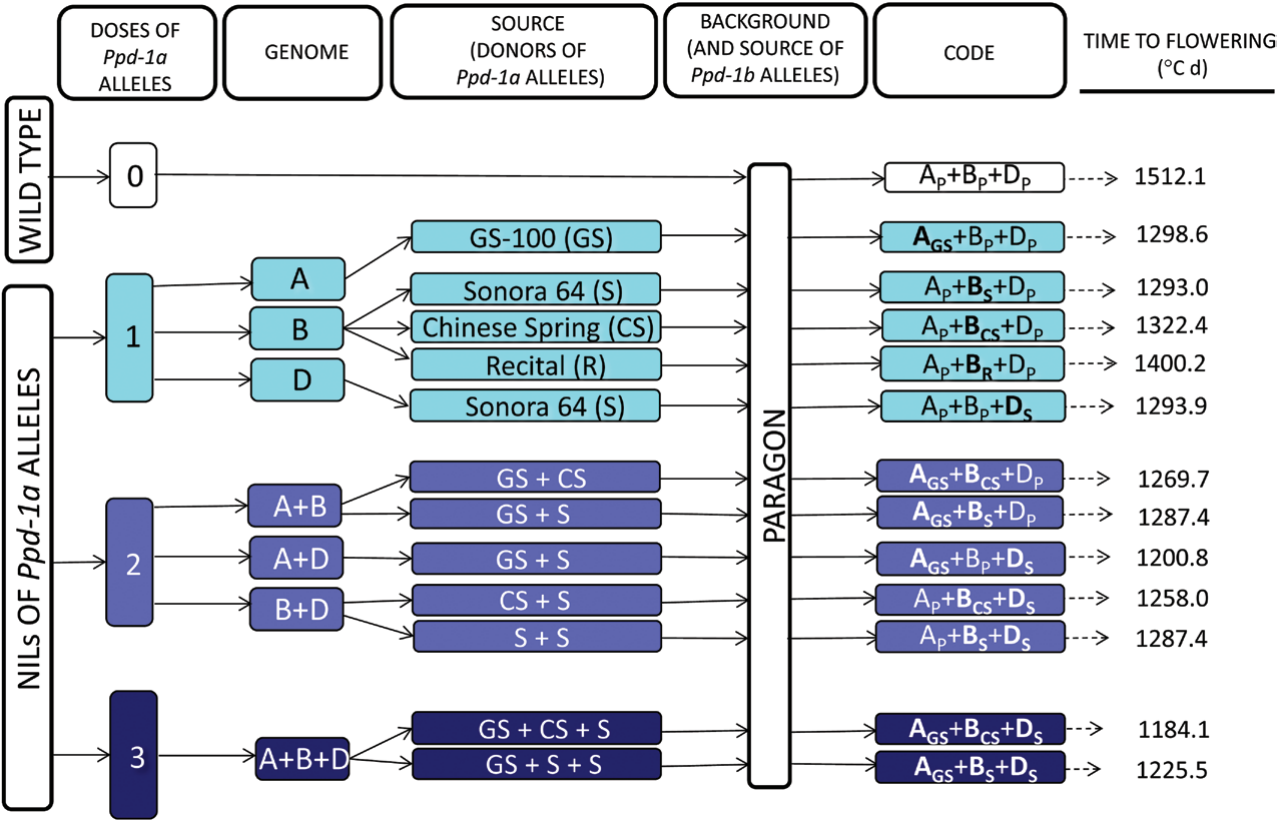
Scheme of genotypes used in this study. Paragon, a spring wheat carrying no insensitivity alleles and its near isogenic lines (NILs) with one, two, or three doses of *Ppd*-*1a* alleles introgressed. The scheme indicates the genome in which photoperiod-insensitive alleles were introgressed, the donors of these *Ppd*-*1a* alleles, and the code followed in this work for naming the different genotypes (it consists of the upper case letters A, B, and D representing the three genomes with a subscript indicating the source of the *Ppd-1a* alleles). The subscript ‘P’ indicates that the *Ppd* alleles were sensitive (*Ppd-1b* alleles) and belong to Paragon (in plain text), while the other subscripts refer to the different donors of *Ppd-1a* alleles (in bold type). The last column shows the thermal time to flowering (averaged across seasons) for each NIL. Adapted from Ochagavía *et al.* (2017), with permission from Elsevier. (This figure is available in colour at *JXB* online.)

Treatments consisted of Paragon, a spring cultivar strongly sensitive to photoperiod, with *Ppd-1b* alleles in all the three genomes (Winfield *et al.*, 2010; Shaw *et al.*, 2012), and 12 NILs in which photoperiod insensitivity alleles (*Ppd-A1a*, *Ppd-B1a*, and *Ppd-D1a*) were introgressed (alone or in combinations of two or three alleles) in its background (Fig. 1). NILs consisted of (i) five single NILs which carried either *Ppd-A1a*, *Ppd-B1a* (from three different donors), or *Ppd-D1a*; (ii) five double NILs with *Ppd-1a* alleles introgressed in two genomes simultaneously; and (iii) two triple NILs with *Ppd-1a* alleles on all three genomes. The sources (donors) of the introgressed insensitivity alleles were genotypes ‘GS-100’ (for chromosome 2A, carrying a 1027 bp promoter deletion; Wilhelm *et al.*, 2009), either ‘Sonora64’, ‘Chinese Spring’, or ‘Recital’ (for chromosome 2B, characterized by three intact copies in tandem, one truncated copy and three intact copies in tandem, and two intact copies in tandem, respectively; Beales *et al.*, 2007), and ‘Sonora64’ (for chromosome 2D, carrying a 2089 bp promoter deletion; Beales *et al.*, 2007) (Fig. 1). Photoperiod insensitivity alleles were introgressed in the background of the recurrent parent, Paragon, by crossing with the designated donors of insensitivity alleles followed by backcrossing to Paragon at least to BC_6_.

Genotypes were arranged in a complete randomized design with a different number of replicates (ranging from one to five replicates, depending on availability of grains) in 2012/13. Only in two out of the 13 genotypes grown in this first growing season were the available grains so scarce that we could only afford to sow a single field plot (and then there was only one replicate). These were A_P_+**B**_**R**_+D_P_ (Paragon with a single insensitive allele from Recital in chromosome 2B) and **A**_**GS**_**+B**_**S**_**+D**_**S**_ (the triple insensitive NIL with insensitive alleles from GS-100 in chromosome 2A and from Sonora 64 in chromosomes 2B and 2D). As we multiplied the grains and produced our own stock in the first growing season, in the second experiment carried out in the 2013/14 growing season the treatments were arranged in a completely randomized block design with three replicates. Plot size was always of six rows (0.20 m apart) wide and 4 m long.

### Measurements and analyses

From seedling emergence onwards, data were taken periodically for (i) leaves which had appeared (to use it as an alternative to thermal time as the independent variable to analyse the dynamics of primordia initiation); and (ii) cumulative number of primordia initiated in, as well as the stage of development of, the apex.

For determining leaf appearance, three plants per plot that were at the expected plant density in its surrounding area and that were representative of the cohort of emergence were selected, labelled, and then monitored. In each sampling, the number of leaves which appeared in the main shoot (Haun, 1973) was recorded. Then the cumulative number of leaves which appeared was plotted against thermal time and the reciprocal of the slope of the relationship was the phyllochron for that particular treatment. Thermal time was calculated with mean air temperature [i.e. assuming a base temperature of 0 ºC and that the maximum temperature (*T*max) was never above the optimum temperature]. Furthermore, we estimated the photothermal time as Σ*L* (*T*_L_–*T*_B_), where *L* is daylength as a fraction of 24 h, *T*_L_ is the average temperature during daylight hours, and *T*_B_ is the base temperature (Masle *et al.*, 1989). However, as we found similar results with both estimations of duration of phases, we presented the data based on thermal time, which is the most frequent approach in the literature.

**Fig. 2.**
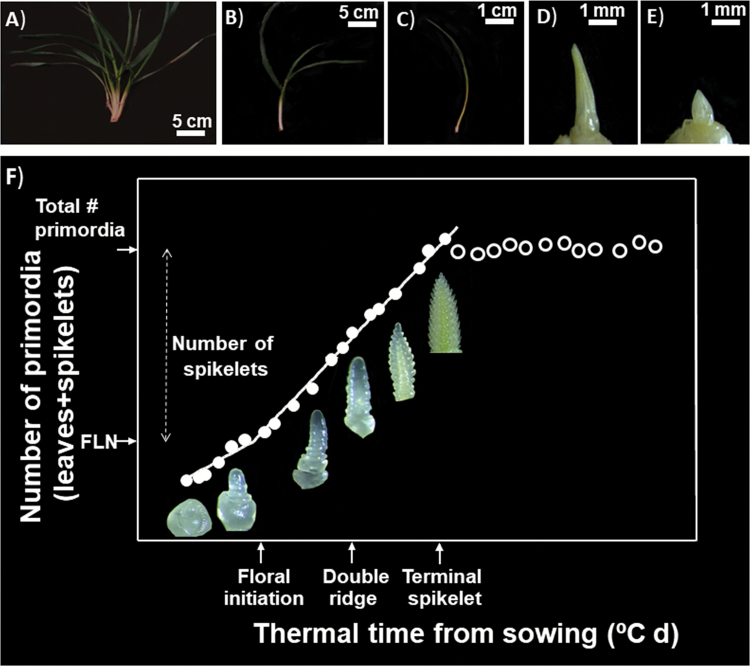
Schematic diagram of apex dissection (top panels A–E): expanded leaves are removed first (A–C) and then the unexpanded leaves covering the apex are also removed under a binocular microscope (D and E). Once the apex is reached, (i) its stage is determined, according to the scale proposed by Waddington *et al.* (1983), until the terminal spikelet (pictures inside bottom panel, F), and (ii) the number of primordia (leaves+spikelets) is counted. Then the number of primordia is plotted against thermal time in order to analyse the dynamics of primordia initiation as illustrated, within the period from seedling emergence and terminal spikelet (filled symbols; F). Final leaf number (FLN) is determined counting the leaves which have appeared following the scale of Haun (1973). The total number of primordia is the average of the values measured in a number of samples (>10) taken from the terminal spikelet to anthesis (open symbols; F). The number of spikelets is calculated as the difference between the total number of primordia and FLN. Floral initiation is estimated *a posteriori* (there is no morphological evidence in the dissected apex for this critical stage) as when the first reproductive primordium was initiated, when the total number of primordia exceeded FLN by one. (This figure is available in colour at *JXB* online.)

For determination of the stages of apex development and quantification of the number of primordia initiated over time, one representative plant per plot was randomly sampled at frequent intervals (from once to three times a week, depending on temperatures). All in all there were 27 plants sampled and dissected from each plot from seedling emergence to the 1–2 weeks after anthesis. The first date of dissecting sampling presented a photoperiod of 9.53 h and 9.00 h, whilst the last sampling date had photoperiods of 14.48 h and 14.22 h, in both cases for the first and the second growing season, respectively. In all these cases, sampled plants were taken to the lab and dissected under a binocular microscope (Leica MZ 7.5, Leica Microsystems, Heerbrugg, Switzerland). We first determined the developmental stage of the apex (according to the scale produced by Waddington *et al.*, 1983) and then counted the total number of primordia (leaf and spikelet), including the number of leaves already grown that were removed to dissect the apex (Fig. 2, top panels). With these data, the dynamics of leaf and spikelet primordia initiation were analysed by plotting the number of primordia against thermal time from sowing and fitting bilinear regressions (Fig. 2, bottom panel). The first phase in which initiation of leaf primordia took place was much slower than the second phase when spikelet primordia were initiated. The bilinear model we fitted had a fixed intercept of four primordia (i.e. corresponding to the four leaves initiated during grain filling in the mother plant and, therefore, already developed in the embryo of the grains) and a fixed change in slope at the timing of floral initiation. Plastochron (i.e. the thermal time elapsed between the initiation of two successive primordia in the apex) was estimated as the reciprocal of the rate of primordia initiation. Thus, there were two distinct plastochrons estimated from the bilinear regressions: the first corresponding to the leaves was much longer than the second which corresponds to spikelet plastochron. Timing of floral initiation was finally determined when the total number of primordia of the apex exceeded the FLN on the main shoot by one primordium (González *et al.*, 2002), reassessed using the fitted model and the FLN. Double ridge and terminal spikelet stages were determined under a binocular microscope in the dissection process according to the scale developed by Waddington *et al.* (1983) (Fig. 2).

From the analysis of the dynamics of primordia initiation described above, it would not be possible to establish whether any eventual effect of *Ppd* genes on the rates of leaf or spikelet initiation was direct or simply a reflection of the effects of these alleles on other developmental processes. We therefore analysed the relationship between leaf and spikelet primordia and number of emerged leaves (i.e. phyllochrons) instead of thermal time, so that effects of *Ppd* genes beyond those that they may exert on phyllochron could be uncovered. Due to the constitutive difference in rates of primordia initiation of leaves and spikelets, this relationship presented a bilinear trend as well, and was fitted with a bilinear model. In this case, the model used a fixed intercept of six primordia, because the origin in this relationship represents seedling emergence, and seedlings presented two leaf primordia in addition to those present in the embryo, which were initiated between sowing and seedling emergence (Hay and Kirby, 1991).

As dissections continued after the terminal spikelet for determining the developmental dynamics of individual florets (see companion paper, Prieto *et al.*, 2018), the number of spikelets per spike was counted in each of these post-terminal spikelet dissections for each plot, and the final number of spikelets per spike was calculated as the average of all these values (Fig. 2). To validate this procedure, we also determined the number of spikelets per spike in a larger sample taken at anthesis. This sample consisted of all plants in 0.5 m of a central row, which had been labelled after seedling emergence guaranteeing that the plant density and uniformity were those ideally expected in the sample area and its borders. In these plants, the spikelets were counted on main shoot and tiller spikes separately.

## Results

In order to validate our determinations made from individual plants of each plot sampled repeatedly through the growing season, we compared the number of spikelets per spike determined through the dynamics of primordia initiation with the same variable measured in the plot sample taken at anthesis. We observed that the number of spikelets per spike determined averaging the many individual plants we sampled from terminal spikelet to anthesis was the same value that we determined in the larger sample taken at anthesis (see Supplementary Fig. S1 at *JXB* online).

**Fig. 3.**
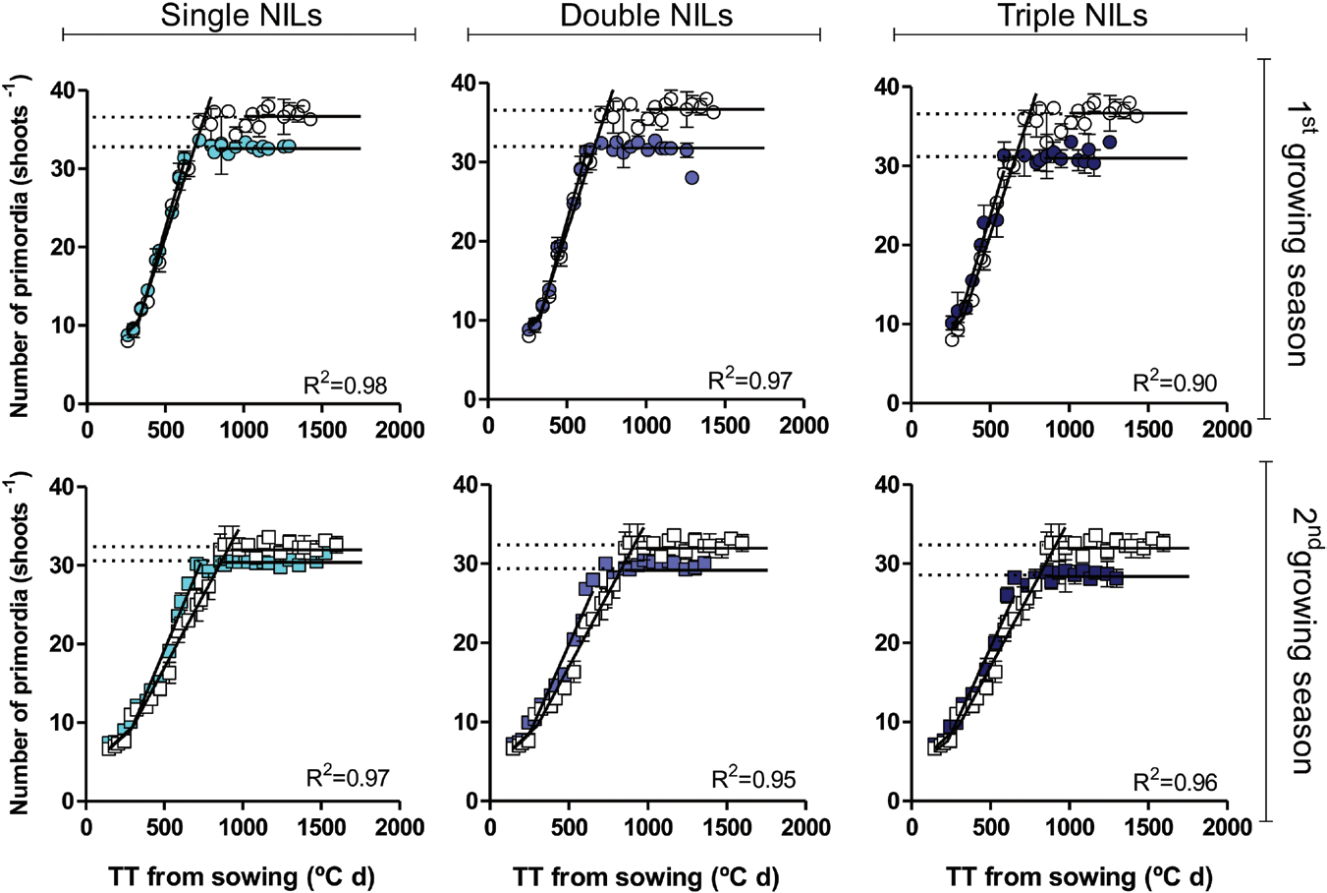
Relationship between the number of primordia (leaves+spikelets) and thermal time for NILs (filled symbols) with single, double, or triple doses of *Ppd-1a* alleles introgressed in the background of the wild-type Paragon (open symbols) in the first (top panels) and second (bottom panels) growing seasons. The *R*^2^ values indicated in the panels correspond to the relationships of the NILs with *Ppd-1a* alleles (*R*^2^=0.94 and 0.95 for Paragon in the first and second growing seasons, respectively); these coefficients of determination were always highly significant (*P*<0.01). (This figure is available in colour at *JXB* online.)

This was also consistent with the fact that in all cases, there was a rather constant value of the total number of primordia in the many measurements made after the terminal spikelet (with a small variation that can be expected as at each sampling time a spike of a different plant was quantified; see the plateau in Fig. 3), providing confidence in the number of spikelets per spike determined at the terminal spikelet stage and quantified as the average of all dissections made from then on.

The rate of primordia production and its reciprocal (plastochron) were calculated from plotting the total number of primordia against thermal time for each particular genotype and in each growing season. The relationships are illustrated for the average of all NILs with single, double, or triple doses of *Ppd-1a* alleles always compared with wild-type Paragon with *Ppd-1b* alleles in all three genomes (Fig. 3), but the reciprocal of both slopes (leaf and spikelet plastochrons) are offered for each individual case (Table 2). Spikelets were initiated at a much higher rate than leaves (Fig. 3), and therefore leaf plastochron was much longer than spikelet plastochron in all cases (Table 2). Averaging across all genotypes and both growing seasons, the initiation of spikelet primordia was almost 67% faster than that for leaf primordia.

**Table 2.** Leaf and spikelet plastochrons, derived from the slopes of the bilinear relationships between number of primordia (leaves+spikelets) and thermal time (whose coefficients of determination are also included) for each of the NILs and the wild-type Paragon, A_P_+B_P_+D_P_ (as well as for the average of all NILs with the same dose of *Ppd-1a* alleles) in both growing seasons

Genotype	First growing season			Second growing season		
	Leaf plastochron (ºCd per leaf)	Spikelet plastochron (ºCd per spikelet)	*R* ^2^	Leaf plastochron (ºCd per leaf)	Spikelet plastochron (ºCd per spikelet)	*R* ^2^
A_P_+B_P_+D_P_	48.8 ± 1.7	16.3 ± 1.4	0.94	54.3 ± 1.4	26.9 ± 1.2	0.95
**A**_**GS**_+ B_P_+D_P_	**56.0 ± 0.6**	**13.9 ± 0.6**	0.96	55.2 ± 1.9	**19.9 ± 2.0**	0.89
A_P_+**B**_**CS**_+D_P_	46.5 ± 1.5	14.0 ± 1.5	0.94	**57.1 ± 1.2**	24.2 ± 1.5	0.94
A_P_+**B**_**S**_+D_P_	**53.5 ± 0.9**	**13.9 ± 0.8**	0.95	53.7 ± 1.3	**22.7 ± 1.5**	0.93
A_P_+**B**_**R**_+D_P_	47.7	16.3	0.99	**59.1 ± 1.4**	**20.7 ± 1.4**	0.93
A_P_+B_P_+**D**_**S**_	**54.4 ± 0.9**	**13.7 ± 0.8**	0.96	55.6 ± 1.2	**20.4 ± 1.4**	0.95
${\bar{\text{X}}}_{\text{Single}}$	51.6 ± 1.9	**14.3 ± 0.5**		56.1 ± 0.9	**21.6 ± 0.8**	
**A**_**GS**_ **+B**_**CS**_+D_P_	**64.5 ± 0.8**	15.3 ± 0.8	0.95	53.6 ± 1.3	25.0 ± 1.5	0.95
**A**_**GS**_+B_P_+**D**_**S**_	46.1 ± 1.5	13.6 ± 1.4	0.88	**59.4 ± 1.4**	**23.1 ± 1.4**	0.95
A_P_+**B**_**CS**_**+D**_**S**_	**60.2 ± 1.0**	**13.5 ± 1.0**	0.95	52.7 ± 1.6	**20.9 ± 1.8**	0.93
**A**_**GS**_ **+B**_**S**_+D_P_	**54.6 ± 1.3**	14.6 ± 1.2	0.94	**57.5 ± 1.4**	**23.2 ± 1.9**	0.90
A_P_+**B**_**S**_**+ D**_**S**_	**59.5 ± 0.7**	**13.9 ± 0.6**	0.96	**57.7 ± 1.6**	**20.0 ± 1.7**	0.94
${\bar{\text{X}}}_{\text{Double}}$	**56.8 ± 3.3**	**14.3 ± 0.3**		56.2 ± 1.3	**22.4 ± 0.9**	
**A**_**GS**_ **+B**_**CS**_ **+D**_**S**_	**52.1 ± 0.8**	**13.8 ± 0.7**	0.93	**71.5 ± 1.8**	**22.9 ± 2.1**	0.90
**A**_**GS**_ **+B**_**S**_ **+D**_**S**_	54.2	16.7	0.87	**61.2 ± 1.3**	**22.5 ± 1.3**	0.96
${\bar{\text{X}}}_{\text{Triple}}$	**53.1 ± 1.1**	15.3 ± 1.5		**66.4 ± 5.1**	**22.7 ± 0.2**	

Values indicate the mean ±SE, with the exception of the two genotypes that were grown in a single repetition in the first growing season (see the Materials and methods). Bold values indicate that differences in plastochrons between NILs and Paragon were larger than the differences between their SEs. The *R*^2^ values were always highly significant (*P*<0.001).

The introgression of *Ppd-1a* alleles reduced the number of primordia initiated. In general, lines produced fewer primordia in the second than in the first growing season (Fig. 3), but in both seasons there was a clear trend to reduce the number of primordia initiated with the increase in the doses of insensitivity alleles (Fig. 3). In general, data points of the NILs having *Ppd-1a* alleles were rather overlapped with those of Paragon, although with a consistent trend to increase the rate of spikelet initiation when photoperiod insensitivity alleles were introgressed. Therefore, the main difference in the final number of primordia between lines with different doses of *Ppd-1a* alleles was explained by the timing of initiation of the primordia endpoint in each case (Fig. 3). Also, the magnitude of the difference in number of primordia between NILs and Paragon was smaller in the second than in the first growing season, mainly because the *Ppd-1a* alleles more clearly accelerated the rate of spikelet primordia initiation in the second season, compensating in part the advance produced in timing of terminal spikelet initiation (Fig. 3; Table 2).

When considering each individual NIL, the bilinear model fitted the data of primordia number versus thermal time extremely well in both growing seasons (*R*^2^=0.87–0.99; *P*<0.01) (Table 2), as was the case when the averages of NILs having the same dose of *Ppd-1a* alleles were analysed (Fig. 3).

There were rather minor and inconsistent effects of *Ppd-1a* alleles on leaf plastochron, while there was a significant and consistent trend for NILs with *Ppd-1a* alleles introgressed to reduce the spikelet plastochron of Paragon (Table 2). The effect on spikelet plastochron seemed to have been stronger for *Ppd-A1a* and *Ppd-D1a* than for the average of *Ppd-B1a* alleles. However, there was also variation depending on the source of the alleles as the *Ppd-B1a* allele introgressed from Chinese Spring only marginally (and not significantly) reduced the spikelet plastochron while the *Ppd-B1a* allele introgressed from Sonora 64 accelerated the rate of spikelet initiation much more strongly (and the results of introgressing the *Ppd-B1a* allele from Recital were inconsistent across growing seasons; Table 2).

**Fig. 4.**
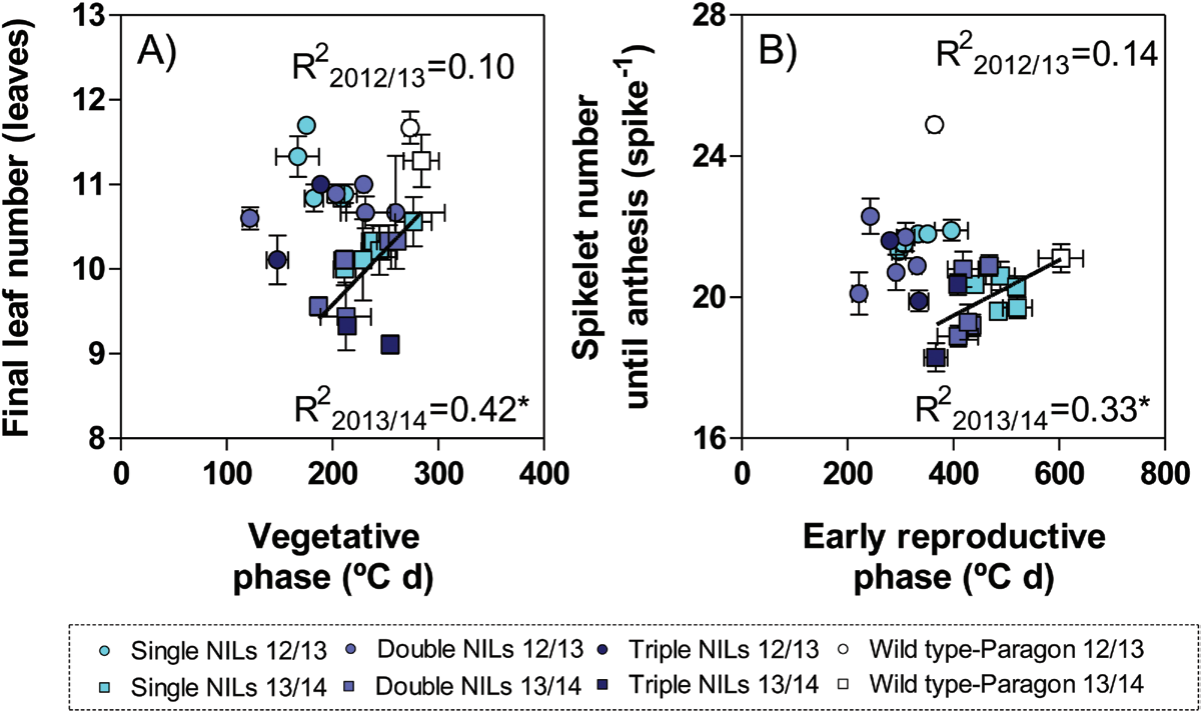
Relationship between the final number of leaves on the main shoot (A) or spikelets in the main shoot spike (B) and the duration of the phases when these organs were initiated (vegetative and early reproductive phases, respectively) for NILs (filled symbols) with different doses of *Ppd-1a* alleles introgressed in the background of the wild-type Paragon, A_P_+B_P_+D_P_ (open symbols), in the first (circles) and second (squares) growing seasons. Symbols for the NILs are shown as a ‘heat map’ representing the number of insensitive alleles; NILs with single, double, and triple doses of *Ppd-1a* alleles are shown with symbols with light, medium, and dark filling, respectively. (This figure is available in colour at *JXB* online.)

Thus, differences in the final number of primordia between different NILs and wild-type Paragon were related to the effects of the photoperiod insensitivity alleles on reducing the duration of the vegetative and early developmental phases, although the relationship was significant only in the second growing season (Fig. 4); whilst there was no solid relationship whatsoever between FLN and leaf plastochron (Supplementary Fig. S2, top panels) or between spikelets per spike and spikelet plastochron (Supplementary Fig. S2, bottom panels). This lack of a clear relationship between the final number of organs and their rate of initiation when comparing NILs for *Ppd-1* genes simply reflects that these genes did not affect plastochron when they affected the rate of phasic development of the leaf and spikelet initiation phases (Supplementary Fig. S3).

It is worth noting that in the second growing season (when the relationships were significant), the change in number of leaves initiated from seedling emergence (before then plants do not respond to photoperiod) to floral initiation was proportional to the reduction in duration of the vegetative phase, while this was not the case for the number of spikelets initiated during the early reproductive phase and the duration of this phase. If we assume that at the time of seedling emergence seedlings already had six leaf primordia initiated, the number of leaf primordia initiated from then to floral initiation ranged from ~3 (in the triple insensitive NILs) to 5 in Paragon, while the duration of the vegetative phase ranged from <200 °Cd to ~300 °Cd (Fig. 4A). The number of spikelets per spike, on the other hand, ranged from ~18 (in one of the triple insensitive NILs) to ~21 in Paragon, while the duration of the early reproductive phase ranged from <400 °Cd to >600 °Cd (Fig. 4B). The reason for this was the differential effect of photoperiod insensitivity alleles on leaf and spikelet plastochron (the latter was reduced, partially compensating for the reduction these alleles produced in the early reproductive phase).

The strength of the reduction in the number of primordia in the main shoots depended partly on the doses of *Ppd-1a* alleles introgressed and partly on the specific allele considered. There seemed to be a general trend for NILs with higher doses of insensitivity alleles to reduce the duration and the final number of primordia initiated in that period, but with variation depending on the specific allele being considered (Fig. 4). The genotype with the lowest leaf and spikelet numbers was one of the NILs with triple introgression (**A**_**GS**_**+B**_**CS**_**+D**_**S**_) in both growing seasons (10.1 ± 0.3 and 9.1 ± 0.1 leaves and 19.9 ± 0.3 and 18.3 ± 0.4 spikelets per spike for the first and second growing seasons, respectively), while the genotype with the highest numbers was the wild-type Paragon (11.7 ± 0.2 and 11.3 ± 0.3 leaves and 24.9 ± 0.2 and 21.1 ± 0.4 spikelets per spike for the first and second growing seasons, respectively).

**Fig. 5.**
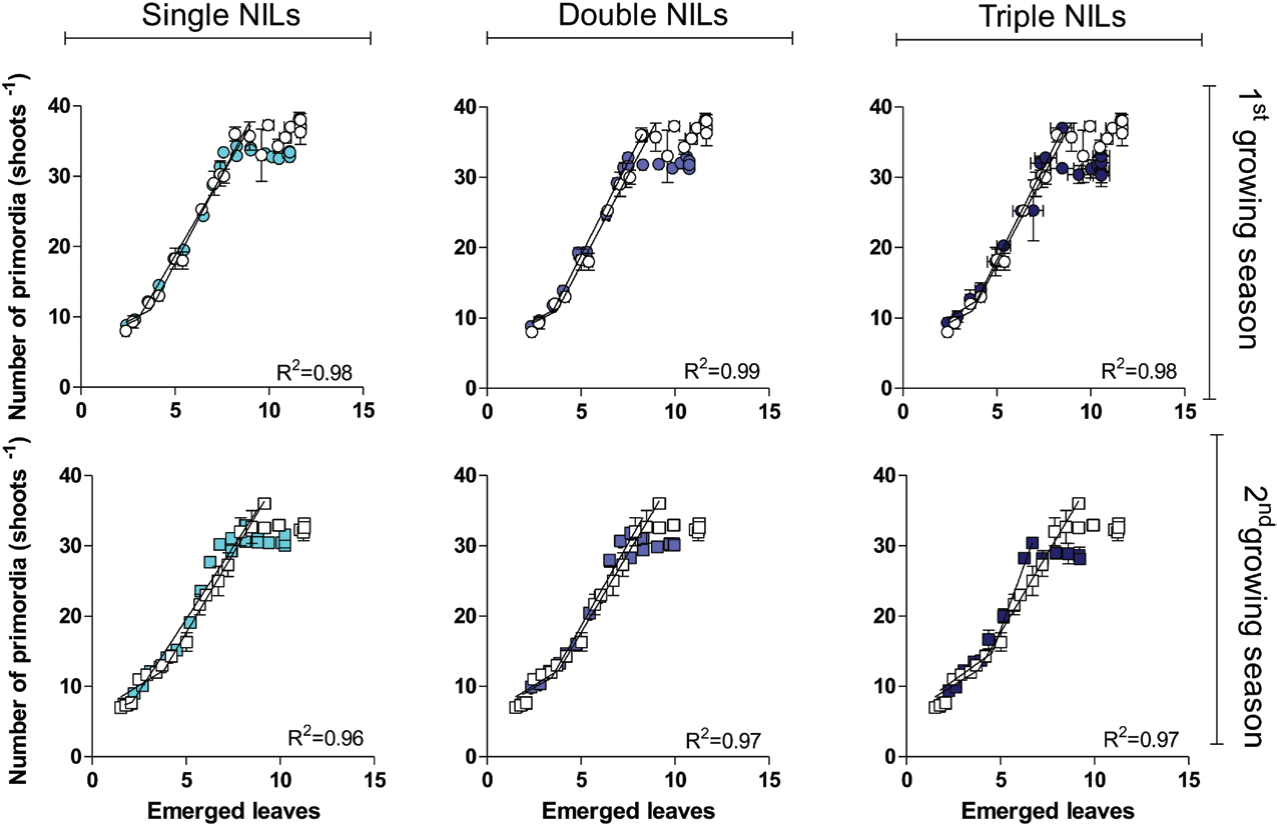
Relationship between the number of primordia (leaves+spikelets) and the number of emerged leaves for different NILs (filled symbols) with single, double, or triple doses of *Ppd-1a* alleles introgressed in the background of the wild-type Paragon (open symbols) in the first (top panels) and second (bottom panels) growing seasons. The *R*^2^ values indicated in the panels correspond to the relationships of the NILs to *Ppd-1a* alleles (*R*^2^=0.985 and 0.989 for Paragon in the first and second growing seasons, respectively); these coefficients of determination were always highly significant (*P*<0.01). (This figure is available in colour at *JXB* online.)

The primordia production rate was calculated in phyllochrons as well, based on the bilinear regression fitted to the data points of the relationships between the cumulative number of primordia initiated in the apex and the number of emerged leaves on the main shoots (Supplementary Table S1), as illustrated for the average of all NILs with single, double, or triple doses of *Ppd-1a* alleles versus Paragon (Fig. 5). On average, 1.7 leaves and 5.6 spikelets were initiated per phyllochron. Photoperiod insensitivity alleles did not clearly affect the co-ordination between primordia initiation and leaf emergence, but spikelet plastochron (in terms of spikelets initiated per phyllochron) tended to be slower in NILs carrying these insensitivity alleles than in wild-type Paragon (Supplementary Table S1). In other words, *Ppd-1a* alelles reduced spikelet plastochron when calculated in thermal time, but the opposite was true when spikelet plastochron was estimated in phyllochrons. Consequently, there was not a consistent effect of the doses of *Ppd-1a* alleles on spikelet plastochron measured in phyllochrons (i.e. as doses increased the spikelet plastochron did not always increase). In addition, no clear effects of the source of *Ppd-1a* alleles was found on spikelet plastochron (Supplementary Table S1).

**Fig. 6.**
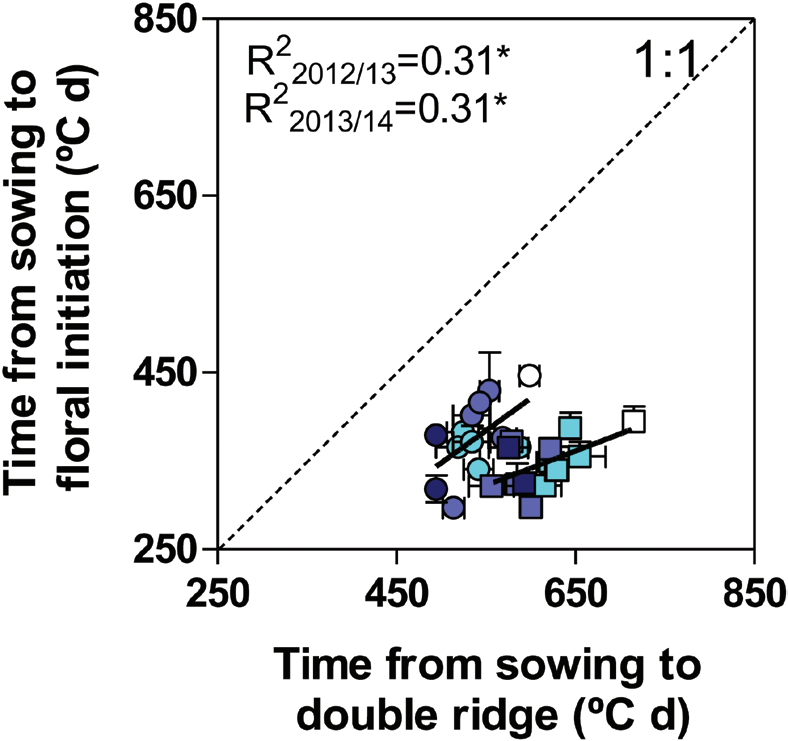
Relationship between periods from sowing to double ridge and to floral initiation for NILs (filled symbols) with different doses of *Ppd-1a* alleles introgressed in the background of the wild-type Paragon, A_P_+B_P_+D_P_ (open symbols), in the first (circles) and second (squares) growing seasons. Symbols for the NILs are shown as a ‘heat map’ representing the number of insensitive alleles; NILs with single, double, and triple doses of *Ppd-1a* alleles are shown with symbols with light, medium, and dark filling, respectively. The dotted line stands for the 1:1 ratio, where data points should be if double ridge was actually floral initiation. (This figure is available in colour at JXB online.)

Double ridge occurred later than floral initiation in all genotypes in both seasons (Fig. 6, with all data points clearly above the 1:1 ratio). *Ppd* insensitivity alleles reduced the period from sowing to the double ridge stage, and the general trend was a stronger reduction with increasing doses of insensitivity alleles. Thus, when averaged across NILs of the same number of *Ppd-1a* alleles, NILs with single, double, and triple doses of insensitivity alleles reduced time to the double ridge by 10.5, 13.5, and 19.5%, respectively, with respect to Paragon. There was a positive trend (significant only to a probability <5%) between duration of time from sowing to double ridge and that to floral initiation, indicating that the advance in development produced during the vegetative phase by the introgression of insensitivity alleles was maintained through the early reproductive phase until the appearance of the double ridge. However, the effects of *Ppd-1a* alleles on the specific difference between true floral initiation and the appearance of a double ridge in the apex were not consistent. The lack of consistency was not only revealed by the relatively low coefficients of determination but also by the fact that in the first growing season the relationship tended to converge with the 1:1 line, while in the second season the trend was to diverge slightly (Fig. 6). In other words, there was not a consistent trend for insensitive lines to exhibit a more coincident timing of floral initiation and double ridge than the sensitive lines.

## Discussion

While *Ppd-1a* alleles accelerated the rate of phasic development during the vegetative period of leaf initiation, they did not affect the rate of leaf initiation. This finding agrees with those of previous studies in which leaf plastochron was not affected by the action of *Ppd-1a* alleles (e.g. Snape *et al.*, 2001; Whitechurch and Slafer, 2002). Consequently, *Ppd-1a* alleles affected FLN due to reducing the duration of the vegetative phase (e.g. Miglietta, 1989; Slafer and Rawson, 1994*b*), disregarding whether the length of the phase was determined in thermal time or in phyllochrons. This is because, in agreement with previous studies, the rate of appearance of early leaves seems insensitive to photoperiod (Slafer and Rawson, 1997) and unaffected by photoperiod insensitivity genes (González *et al.*, 2005; Ochagavía *et al.*, 2017), although exceptions can be found, at least when grown in controlled conditions (Pérez-Gianmarco *et al.*, 2018). This parallelism is the main reason why the effects of daylength and/or photoperiod insensitivity genes on wheat development can frequently be estimated through their effects on FLN (e.g. Brooking *et al.*, 1995; Slafer and Rawson, 1995; Brooking and Jamieson, 2002; Whitechurch and Slafer, 2002; González *et al.*, 2005).

Spikelets were generated at a faster rate than the leaves, as has been reported repeatedly in the literature (e.g. Kirby, 1974, 1990; Delécolle *et al.*, 1989; Miralles and Richards, 2000). Unlike what we described for the dynamics of leaf primordia initiation, *Ppd-1a* alleles accelerated the spikelet initiation rate (reducing spikelet plastochron) with respect to the photoperiod-sensitive variety Paragon (an analogous effect to that of winter wheats; González *et al.*, 2002). This increased rate of spikelet initiation produced a partial trade-off with the effects of these alleles on the duration of the spikelet initiation phase. The consequence of this partial trade-off was a relatively smaller reduction in number of spikelets per spike due to the introgression of alleles conferring photoperiod insensitivity. This genetic effect is in line with a slight increase in spikelet initiation rates observed when plants of a sensitive cultivar are grown under longer photoperiods, which partially compensates for the effects on reducing the duration of the early reproductive phase (see discussion in Slafer and Rawson, 1994*b*). In the present study, *Ppd* insensitivity alleles reduced the number of spikelets per spike (as was frequently found elsewhere, e.g. Worland *et al.*, 1998; Snape *et al.*, 2001; González *et al.*, 2005), but the magnitude of the reduction was not commensurate with the reduction in duration of the early reproductive phase. This is relevant as advancing time to anthesis through these genes would bring about a proportionally smaller reduction in spikelets per spike and therefore adaptation would be improved, with only marginal potential losses in this yield component. *Ppd-D1a* and *Ppd-A1a* had the fastest spikelet initiation rates (the shortest spikelet plastochrons), whilst *Ppd-B1a* alleles presented a rate of spikelet initiation slower than those of the lines with the other insensitivity alleles (though still faster than the control Paragon). It is worth noting that the proposed functional polymorphisms for *Ppd-D1a* and *Ppd-A1a* both involve a promoter deletion, with both deletions overlapping (Wilhelm *et al.*, 2009), whereas *Ppd-B1a* insensitivity is probably due to increased copy number (Díaz *et al.*, 2012). This ranking in strength for these *Ppd-1a* alleles agrees with the conclusions of Scarth *et al.* (1985) and González *et al.* (2005). In fact, the effect of *Ppd-B1a* alleles on spikelet plastochron did actually vary with the source of the allele introgressed in the background of Paragon. This might explain the controversy in the literature when in some cases it was reported that *Ppd-B1a* alleles increased the rate of spikelet initiation under a short photoperiod more than *Ppd-D1a* (Whitechurch and Slafer, 2002).

When plastochrons were quantified based on another developmental trait such as phyllochron, the effects of *Ppd-1a* alleles on spikelet plastochron calculated in thermal time disappeared. This means that the effects of these alleles on the rate of spikelet initiation actually reflected those on the rate of leaf appearance (Ochagavía *et al.*, 2017), reinforcing the idea that the co-ordination between primordia initiation and leaf appearance is relatively conservative (Kirby, 1990; Miralles *et al.*, 2001). In fact, the model proposed by Kirby (1990) for the co-ordination between primordia initiation and the dynamics of leaf appearance predicted that spikelet plastochron was reduced (and the rate of spikelet initiation was increased) with reductions in FLN, and our results agree with that prediction as the introgression of *Ppd-1a* alleles reduced FLN (see discussion above).

*Ppd* insensitivity alleles advanced the timing of double ridge, as they accelerated the rates of pre-anthesis phasic development. However, the fact that double ridge occurred well after floral initiation (as recognized several times in the past: Delécolle *et al.*, 1989; Kirby, 1990) was not eliminated by the introgression of these alleles. The introgression of *Ppd-1a* alleles did not consistently affect the difference between true floral initiation and double ridge, indicating that differences in the early reproductive phase (from floral initiation to terminal spikelet; Ochagavía *et al.*, 2017) caused by these alleles were not mediated by differences in the period between floral initiation and double ridge.

We concluded that *Ppd-1a* alleles: (i) did not affect leaf initiation rate and therefore reduced FLN in parallel with reductions imposed on duration of the vegetative phase; but (ii) accelerated the spikelet initiation rate, partially compensating for their effect of shortening the length of the early reproductive phase. This caused a decrease in the number of spikelets per spike that was less than proportional to the reduction in duration of the phase. The magnitude of the effects depended not only on the doses and genome involved, but also on the source of a particular allele considered (in this study *Ppd-B1a*), which may well be the cause of the conflict in the literature when rankings of strengths are produced from different materials and opens up potential for selecting the best homoeoallelic combinations of *Ppd-1* for reducing or delaying time to anthesis (depending on the adaptive target), while maximizing spikelet number.

## Supplementary data

Supplementary data are available at *JXB* online.

Fig. S1. Relationship between the number of spikelet primordia determined as the average and that counted in many plants at anthesis for NILs and wild-type Paragon in both growing seasons.

Fig. S2. Relationship between leaf plastochron and final leaf number and between spikelet plastochron and spikelet number for NILs and wild-type Paragon in both growing seasons.

Fig. S3. Relationships between the duration of the vegetative phase and leaf plastochron and that of the early reproductive phase and spikelet plastochron for NILs and wild-type Paragon in both growing seasons.

Table S1. Leaf and spikelet plastochrons for each NIL and the wild-type Paragon in both growing seasons.

Supplementary Material

## Abbreviations:

FLN: final leaf number
NIL: near isogenic line.
