# Supplementary material for: Dynamics of leaf and spikelet primordia initiation in wheat as affected by Ppd-1a alleles under field conditions
Source: J Exp Bot. 2018 Mar 17;69(10):2621–31. doi: 10.1093/jxb/ery104 (PMC5920321; doi:10.1093/jxb/ery104)
Supplement: Supplementary Material [file ery104_suppl_supplementary_material.pdf]

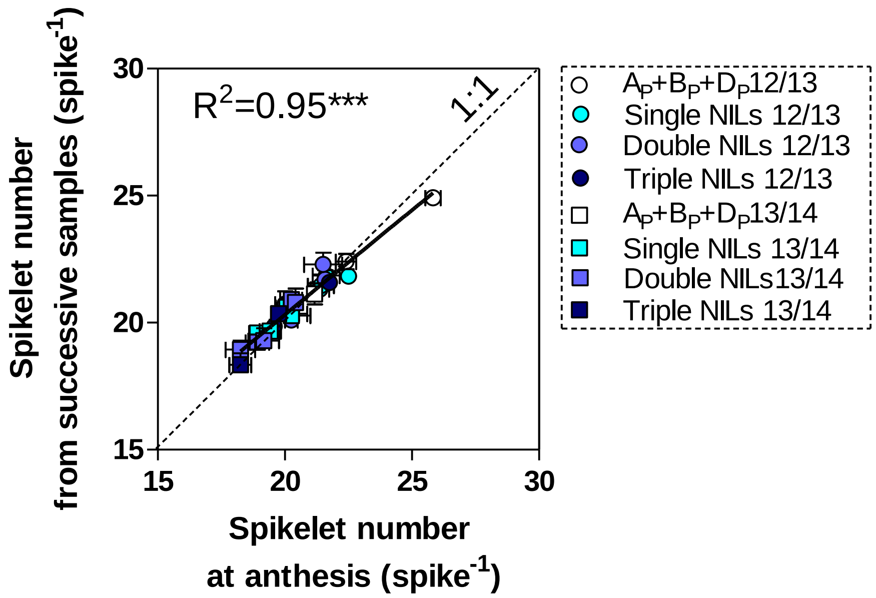

Fig. S1. Relationship between the number of spikelet primordia determined as the average from successive samples of individual plants taken from terminal spikelet to anthesis and that counted simultaneously in many plants from a larger sample taken at anthesis for NILs with single, double or triple doses of *Ppd-1a* alleles introgressed in the background of the wild type-Paragon, $A_P+B_P+D_P$ in both growing seasons.

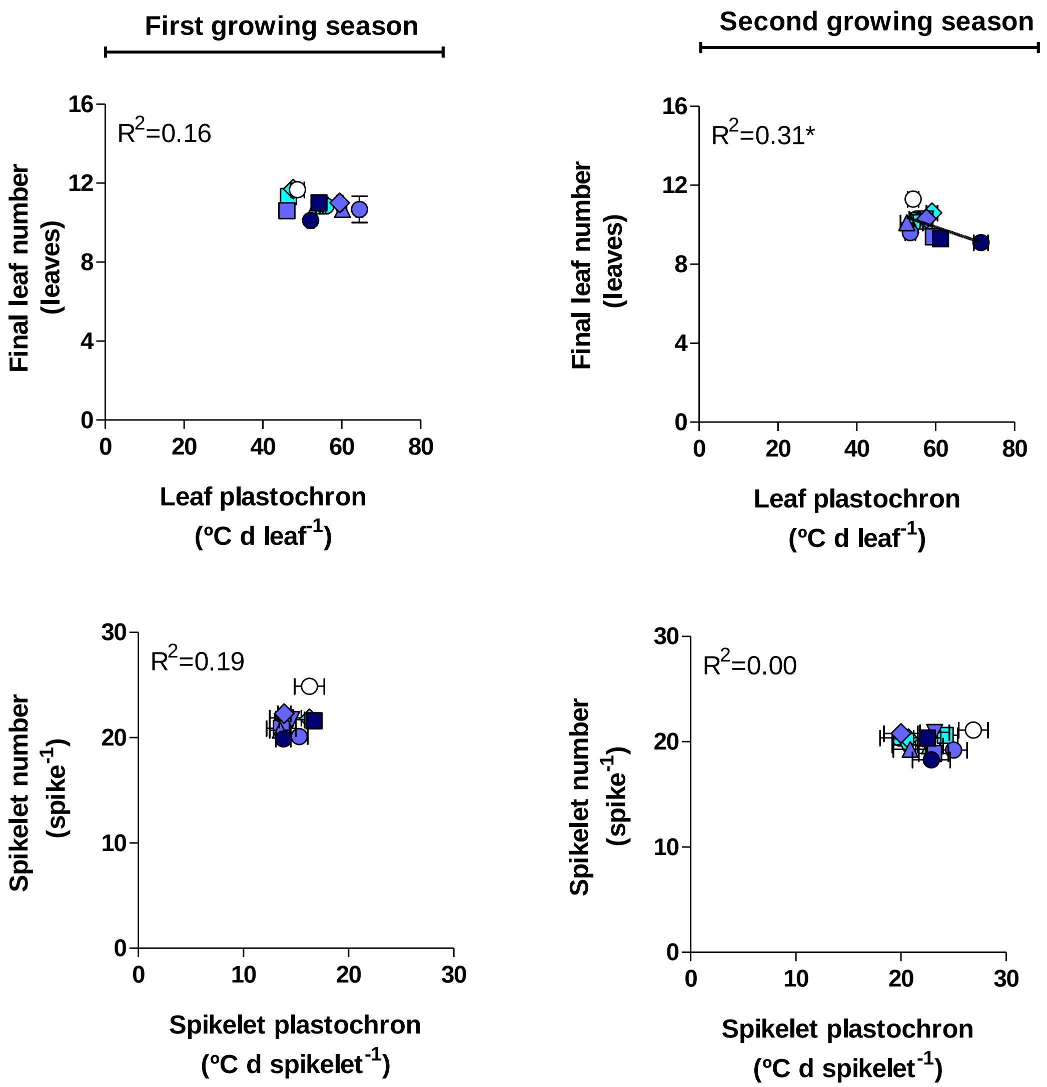

Fig. S2. Relationship between leaf plastochron and final leaf number (top panels) and between spikelet plastochron and spikelet number (bottom panels) for NILs with single, double or triple doses of *Ppd-1a* alleles introgressed in the background of the wild type-Paragon ($A_P+B_P+D_P$) in both growing seasons.

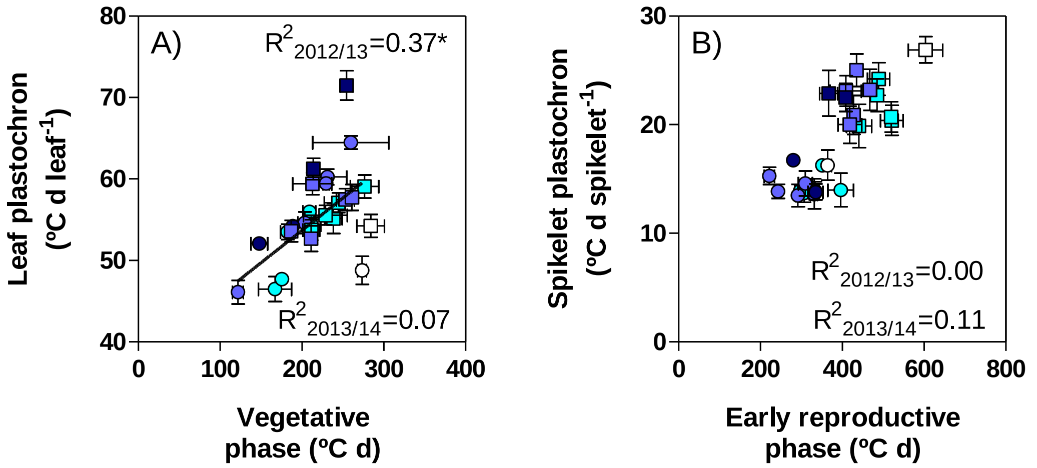

Fig. S3. Relationships between the duration of the vegetative phase and leaf plastochron (A) and that of the early reproductive phase and spikelet plastochron (B) for NILs (closed symbols) with single (light blue), double (blue) or triple (dark blue) doses of *Ppd-1a* alleles introgressed in the background of the wild type-Paragon, $A_P+B_P+D_P$ (open symbols), in the first (circles) and second (squares) growing seasons.

Table S1. Leaf and spikelet plastochrons, derived from the slopes of the bi-linear relationships between number of primordia (leaves+spikelets) and the number of emerged leaves (whose coefficients of determination are also included) for each of the NILs and the wild type-Paragon, A_P+B_P+D_P (as well as for the average o all NILs with the same dose of *Ppd-1a* alleles) in both growing seasons.

	First growing season			Second growing season		
	Leaf primordia (leaf emerged-^{1})	Spikelet primordia (leaf emerged-^{1})	R^2	Leaf primordia (leaf emerged-^{1})	Spikelet primordia (leaf emerged-^{1})	R^2
A_P+B_P+D_P	1.35±0.27	5.09±0.31		1.66±0.16	4.50±0.24	
$\mathbf{A_{GS}}$+B_P+D_P	1.27±0.42	5.10±0.35	0.98	1.68±0.30	**5.59±0.53**	0.97
A_P+$\mathbf{B_{CS}}$+D_P	1.11±0.65	4.53±0.33	0.97	1.64±0.25	5.16±0.42	0.98
A_P+$\mathbf{B_S}$+D_P	1.54±0.25	5.60±0.53	0.98	1.85±0.21	**5.60±0.63**	0.97
A_P+$\mathbf{B_R}$+D_P	2.09	6.09	0.97	1.53±0.44	4.38±0.46	0.95
A_P+B_P+$\mathbf{D_S}$	1.65±0.20	5.70±0.50	0.98	**0.95±0.48**	4.57±0.29	0.97
$\bar{X}_{Single}$	1.53±0.17	5.40±0.27		1.53±0.15	**5.06±0.25**	
$\mathbf{A_{GS}}$+$\mathbf{B_{CS}}$+D_P	**2.14±0.20**	7.26±2.02	0.97	1.90±0.12	**6.15±0.95**	0.98
$\mathbf{A_{GS}}$+B_P+$\mathbf{D_S}$	1.23±0.63	4.89±0.42	0.97	1.90±0.16	**6.59±1.21**	0.97
A_P+$\mathbf{B_{CS}}$+$\mathbf{D_S}$	1.17±0.37	5.09±0.27	0.99	1.59±0.18	**5.43±0.40**	0.98
$\mathbf{A_{GS}}$+$\mathbf{B_S}$+D_P	**2.26±0.26**	**7.19±1.73**	0.95	1.43±0.34	4.67±0.43	0.96
A_P+$\mathbf{B_S}$+ $\mathbf{D_S}$	1.41±0.20	**6.72±0.60**	0.98	1.84±0.22	**6.05±0.76**	0.97
$\bar{X}_{Double}$	1.64±0.23	**6.23±0.52**		1.73±0.09	**5.78±0.33**	
$\mathbf{A_{GS}}$+$\mathbf{B_{CS}}$+$\mathbf{D_S}$	1.35±0.24	5.58±0.19	0.99	1.86±0.17	**7.61±1.23**	0.97
$\mathbf{A_{GS}}$+$\mathbf{B_S}$+$\mathbf{D_S}$	1.75	5.15	0.88	1.99±0.20	**6.96±1.56**	0.96
$\bar{X}_{Triple}$	1.55±0.20	5.37±0.21		**1.92±0.06**	**7.29±0.32**	

Values indicate mean±standard error of the mean (SEM), with the exception of the two genotypes that were grown in a single rep in the first growing season (see Materials and Methods). Bold values indicate that differences in plastochrons between NILs and Paragon were larger than the differences between their SEMs. The R^2s were always highly significant (*P<0.01*).
